# Liver Transcriptome and miRNA Analysis of Silver Carp (*Hypophthalmichthys molitrix*) Intraperitoneally Injected With Microcystin-LR

**DOI:** 10.3389/fphys.2018.00381

**Published:** 2018-04-10

**Authors:** Xiancheng Qu, Menghong Hu, Yueyong Shang, Lisha Pan, Peixuan Jia, Chunxue Fu, Qigen Liu, Youji Wang

**Affiliations:** ^1^National Demonstration Center for Experimental Fisheries Science Education, Shanghai Ocean University, Shanghai, China; ^2^The Key Laboratory of Exploration and Utilization of Aquatic Genetic Resources, Ministry of Education, Shanghai, China; ^3^International Research Center for Marine Biosciences at Shanghai Ocean University, Ministry of Science and Technology, Shanghai, China

**Keywords:** fish, microcystin, toxic effects, RNA-seq, miRNA-seq, integration analysis

## Abstract

Next-generation sequencing was used to analyze the effects of toxic microcystin-LR (MC-LR) on silver carp (*Hypophthalmichthys molitrix*). Silver carps were intraperitoneally injected with MC-LR, and RNA-seq and miRNA-seq in the liver were analyzed at 0.25, 0.5, and 1 h. The expression of glutathione S-transferase (GST), which acts as a marker gene for MC-LR, was tested to determine the earliest time point at which GST transcription was initiated in the liver tissues of the MC-LR-treated silver carps. Hepatic RNA-seq/miRNA-seq analysis and data integration analysis were conducted with reference to the identified time point. Quantitative PCR (qPCR) was performed to detect the expression of the following genes at the three time points: heme oxygenase 1 (HO-1), interleukin-10 receptor 1 (IL-10R1), apolipoprotein A-I (apoA-I), and heme binding protein 2 (HBP2). Results showed that the liver GST expression was remarkably decreased at 0.25 h (*P* < 0.05). RNA-seq at this time point revealed that the liver tissue contained 97,505 unigenes, including 184 significantly different unigenes and 75 unknown genes. Gene Ontology (GO) term enrichment analysis suggested that 35 of the 145 enriched GO terms were significantly enriched and mainly related to the immune system regulation network. KEGG pathway enrichment analysis showed that 18 of the 189 pathways were significantly enriched, and the most significant was a ribosome pathway containing 77 differentially expressed genes. miRNA-seq analysis indicated that the longest miRNA had 22 nucleotides (nt), followed by 21 and 23 nt. A total of 286 known miRNAs, 332 known miRNA precursor sequences, and 438 new miRNAs were predicted. A total of 1,048,575 mRNA–miRNA interaction sites were obtained, and 21,252 and 21,241 target genes were respectively predicted in known and new miRNAs. qPCR revealed that HO-1, IL-10R1, apoA-I, and HBP2 were significantly differentially expressed and might play important roles in the toxicity and liver detoxification of MC-LR in fish. These results were consistent with those of high-throughput sequencing, thereby verifying the accuracy of our sequencing data. RNA-seq and miRNA-seq analyses of silver carp liver injected with MC-LR provided valuable and new insights into the toxic effects of MC-LR and the antitoxic mechanisms of MC-LR in fish.

The RNA/miRNA data are available from the NCBI database Registration No. : SRP075165.

## Introduction

With rapid economic development and population growth, environmental problems have become increasingly serious. A large number of nitrogen and phosphorus have been produced and penetrated water bodies, such as lakes and ponds, resulting in frequent cyanobacterial bloom. The dominant species of algal bloom mainly belong to *Microcystis, Anabaena*, and *Phytophthora*, and some strains can produce secondary metabolites, such as microcystin (MC). MC is a fatal liver toxin that can cause liver cancer (Nishizawa et al., 2000). Francis (1978) first reported that drinking cyanobacterium-contaminated water can induce animal death. Since then, cyanobacteria have been reported to result in large-scale disease and death in birds, fish, livestock, and even humans worldwide. In 1975, drinking water from Sewickley, Pennsylvania, USA, was polluted by toxic cyanobacteria, resulting in acute gastroenteritis that affected about 8,000 people (62% of the local population; Sykora and Keleti, 1981). In 1996, a severe hemodialysis event occurred in Brazil, and at least 60 people died because of the use of MC-contaminated water (Pouria et al., 1998). In summer in 2007, cyanobacterial blooms in Taihu Lake led to the lack of drinking water, seriously affecting the lives of millions of people in Wuxi (Guo, 2007). According to a survey, algal blooms in Taihu Lake and Yangcheng Lake mainly involves *Microcystis* (Ha et al., 2009). Cyanobacterial toxin in drinking water may also be associated with Chinese liver tumor formation (Ueno et al., 1996). MC has also been detected in some edible aquatic animals, and the long-term consumption of these MC-containing aquatic products may harm human health (Magalhães et al., 2001; Amrani et al., 2014). Toxic cyanobacterial blooms in Taihu Lake and Yangcheng Lake have been considered serious water environmental concerns (Zhang et al., 2009).

Thus far, more than 80 MC isomers have been found (Hoeger et al., 2005). Among these isomers, LR-type MCs (MC-LR) exhibit the highest toxicity. The toxic mechanism of MC-LR involves the irreversible inhibition of the activities of protein phosphatase 1 (PP1) and 2A (PP2A) in liver cells, leading to protein overphosphorylation and alteration of the cytoskeleton structure and causing liver hemorrhage and failure (Yoshizawa et al., 1990). Large amounts of reactive oxygen species (ROS) are also produced with oxidative stress, resulting in an irregular fracture of DNA strand and liver cell apoptosis (Chen et al., 2006). MC also affects the growth rate, heart rate, osmotic regulation, and behavior of fish (Wiegand and Pflugmacher, 2005). The antioxidant system of fish comprises superoxide dismutase (SOD), catalase (CAT), glutathione-S-transferase (GST), and glutathione peroxidase (GPx), which belong to phase II detoxification enzymes. When exogenous substances, such as toxins, drugs, contaminants, and carcinogens, enter a fish body, these enzymes can remove excess ROS and toxic substances to maintain the stability of the internal environment (Itoh et al., 1999; Dhakshinamoorthy et al., 2000). The liver is also rich in phase I enzymes. Cytoplasm P (CYP) 450 is one of the key enzymes, including CYP1, CYP2, and CYP3, which play roles in the biotransformation and inactivation of exogenous substances (Goldstein and Faletto, 1993).

Transcriptome sequencing (mRNA-seq) can reveal changes in the expression of differential genes, find different metabolic processes and signaling pathways, and provide accurate digital signals (Bian et al., 2017; Wang et al., 2017). As such, high-throughput sequencing is an effective tool for in-depth studies on transcription. Transcriptome sequencing offers many applications in fish. For example, Song et al. (2015) revealed that 29,526 unigenes are annotated into 329 pathways, and 3,479 differential genes associated with development are aggregated into 50 gene expression profiles. Sequencing results have shown that 37,976 unigenes are annotated into 61 GO terms, 38,172 unigenes are annotated into 275 KEGG pathways, and 38794 simple repeat sequences and 16 polymorphic gene loci are found (Xie et al., 2014). Through the analysis and research of transcriptome data, a large number of unigenes associated with reproduction, growth, and immunization have been identified (Xie et al., 2014).

miRNA is a class of non-coding RNAs that are about 22 nucleotides (nt) in length and implicated in gene expression regulation and many physiological processes, including metabolism, apoptosis, nervous system development, immunoprotection, and cancer pathogenesis (Ambros, 2004; Miska, 2005; Kim et al., 2009). Since the discovery of miRNA (lin-4) in *Caenorhabditis elegans* in 1993 (Lee et al., 1993), an increasing number of studies on the mechanism of miRNA action have been conducted. miRNAs bind to the 3′-untranslated region of a target gene to form RNA-induced silencing complexes, cause post-transcriptional inhibition or deadenylation, and facilitate target gene knockout and degradation (Höck and Meister, 2008). miRNA sequencing can directly reveal differences in miRNA expression levels, discover new or species-specific miRNAs, and predict their target genes, thereby providing an efficient tool for studies on the miRNA function and gene regulation mechanism of species. Huang et al. (2016) completed the miRNA sequencing of loach hips and demonstrated that fibronectin is necessary during angiogenesis and can be regulated by 12 differentially expressed miRNAs. Pathways containing the largest number of miRNA target genes are metabolic pathways, followed by cell cycle pathway, which is essential for the regulation of apoptosis and differentiation (Huang et al., 2016). Xu et al. (2014) conducted an miRNA sequencing of grass carp infected with *Aeromonas hydrophila* and observed a target gene that is involved in most physiological processes, including gene expression, transcription regulation, immune system process, and response to stimulation. Protein kinase and chemokine receptor 7 are frequently implicated in immune function (Xu et al., 2014).

Silver carp (*Hypophthalmichthys molitrix*) is one of the major economic fish feeding on plankton in China and essential for Chinese freshwater aquaculture industry. Silver carp is strongly resistant to MC and implicated in the biological control of algae (Yi et al., 2016). In aquatic animals, toxicity is initially induced by MC through the addition reaction of MC and GSH as catalyzed by the GST gene (Martins et al., 2017). In this study, this gene was considered a reference index to determine the early starting point of the liver response of silver carp intraperitoneally injected with MC-LR. Transcription and miRNA sequencing were conducted, and differentially expressed genes, functional notes, and relevant metabolic pathways were analyzed to further elaborate the toxicity mechanism of MC in fish and the detoxification mechanism of a fish body.

## Materials and methods

### Experimental fish and toxins

Healthy silver carp with an average body weight of 103 ± 5 g (*N* = 18) were collected from a mariculture base in Shanghai Ocean University and acclimated for 3 days in a full aeration tap water (20 ± 2°C) without feeding. The experimental handling and treatment of experimental fish were conducted in accordance with the regulations made by the Institutional Animal Care and Use Committee (IACUC), Shanghai Ocean University (SHOU), and this work was approved by the IACUC of SHOU, Shanghai, China.

MC-LR with a toxin purity of ≥95% was purchased from Beijing Express Technology Co., Ltd. (http://www.express-cn.com/).

### MC-LR injection and extraction of total RNA

The silver carps were divided into two treatments, namely, control and MC-LR injected groups. MC-LR was dissolved in 0.8% NaCl solution at a final concentration of 80 μg mL^−1^. MC-LR was injected intraperitoneally to fish at a dose of 200 μg kg^−1^ body weight. In the control treatment, the silver carps were injected intraperitoneally with an equal volume of 0.8% NaCl solution (Qu et al., 2011). Three silver carps from both the experimental and the control treatments were randomly collected at 0.25, 0.5, and 1 h after exposure and the liver tissue specimens were sampled. After total RNA extractions, samples were dissolved in 100 μL of 0.1% DEPC-ddH_2_O. The OD value and concentration of the extracted total RNA were determined and stored at −80 °C for further analysis.

### Expression of GST gene

Using β-actin as the reference gene, we measured the GST expression through semi-quantitative PCR (Table 1) in both exposure and the control treatments at three sampling points, namely, 0.25, 0.5, and 1 h after exposure. The earliest time point of initiating detoxification in the silver carp after injecton of toxins was selected for the subsequent experiment.

**Table 1:** Primer sequences for PCR amplification.

**Primer**	**Sequence**	**Product size (bp)**
GST F	5′-AGAACGGGCTTTGATTGAC-3′	
GST R	5′-AAGGTTGACAGTATTGTAGGGA-3′	267
β-actin F	5′-ATTGCCGCACTGGTTGTT-3′	
β-actin R	5′-TTTCCCTGTTGGCTTTGG-3′	340

### RNA-seq and *de-novo* assembly

In order to ensure the accuracy of the follow-up experimental analysis, raw data was screened by software SeqPrep and Sickle to acquire a clean data. All clean data were subjected to de-novo assembly for further analysis using Trinity.

### Gene annotations

Clusters of orthologous groups of proteins (COG, http://www.ncbi.nlm.nih.gov/COG/) were used to perform functional annotation, categorization, and protein evolutionary analysis. Gene Ontology (GO, http://www.geneontology.org/) was applied to classify biological processes, molecular functions, and cellular components. Kyoto Encyclopedia of Genes and Genomes (KEGG, http://www.genome.jp/kegg/) was used to analyze complex biological functions, such as metabolic pathways, genetic information transfer, and cellular processes (Li and Durbin, 2009). Thus, the corresponding annotations were obtained, and the significant enrichment conditions were *p* ≤ 0.05 (Xie et al., 2011).

### miRNA–seq quality control

In order to ensure the accuracy of the subsequent analysis, the quality of raw reads obtained from small RNA sequencing was controlled by software SeqPrep and Sickle. The measured small RNAs were annotated by blast according to the Rfam database, where non-miRNA sequences were removed, such as tRNA and rRNA sequences (Burge et al., 2013).

### Expression of known and novel miRNAs and prediction of their target genes

Small RNA sequences were compared with miRNA precursors and mature sequences of the species in the miRBase database by using RNAfold, Bowtie, and miRDeep2. Relevant statistics was mapped to the number of mature sequences, and secondary structures were predicted. The miRNA precursor marker hairpin structure was used to predict new miRNAs (Friedländer et al., 2012). For screening significant differences in miRNA, significance was set at *P* < 0.001. The target genes of all known and novel miRNAs were predicted with MiRanda, and the target genes annotation information was correlated with the miRNAs.

### Conjoint analysis on the RNA-seq and miRNA-seq data and qPCR verification

The transcriptome data was used as a database of miRNA-predicted target genes, and the OrthoMCL method was used to match homologous genes of differentially expressed genes (DEGs) with the target genes of miRNAs. The accuracy of transcriptome sequencing was verified by semi-quantitative PCR and qPCR. Finally, qPCR was used to detect the expression of HO-1, IL-10R1, apoA-I, and HBP2 genes at 0.25, 0.5, and 1 h. Primer sequences for PCR amplification of HO-1, IL-10R1, apoA-I, and HBP2 genes are shown in Table 2.

**Table 2:** Primer sequences for PCR amplification.

**Primer**	**Sequence (5′-3′)**	**Product size (bp)**
HO-1 F	TATGAGATCTACCAAGCGCTAGAGG	
HO-1 R	GTCCAAAGAAGTGCTCCAGGTC	132
IL-10 F	TTCCTGAGGCGTCCTGGTAAAATG	
IL-10 R	TCCTCATCAGAAAGGTCCATGTCC	189
apoA-1 F	TGGCACAGAGTTCAAAGACTACAAG	
apoA-1 R	CGCTTACGCTCCTTGACCAT	283
HBP2 F	GAGTGCTTGCTGTATGACTTGGTT	
HBP2 R	AGCCACATTTTCTCCGGTGATGTAT	165

### Statistical analysis

Data were presented as mean ± standard deviation and analyzed using SPSS 17.0. Before analysis, data normality and homogeneity were checked by Shapiro–Wilk's test and Levene's test, respectively. Significant differences between MC injection treatment and control were compared by Student's *t*-test. Significant differences were denoted by *P* < 0.05.

## Results

### Expression of GST gene

**Figure 1:**
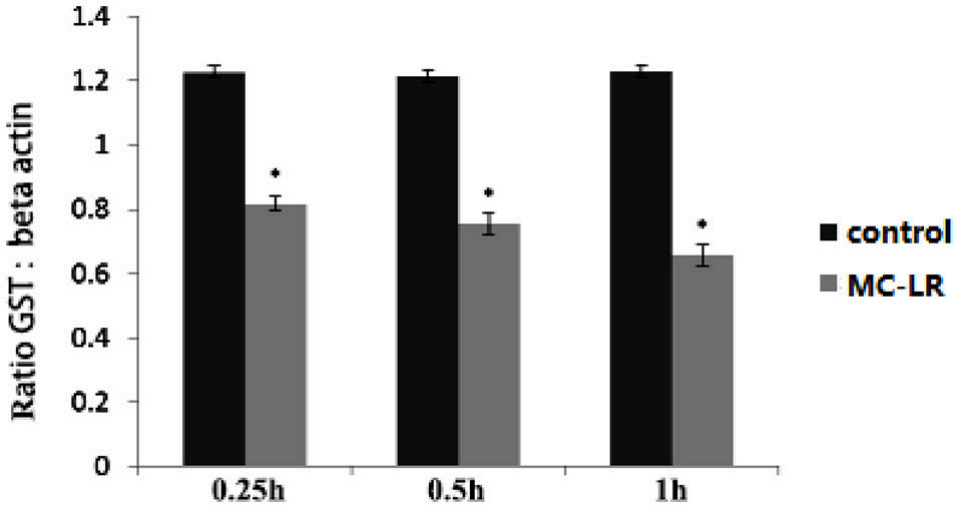
Relative mRNA expression of the GST gene in silver carp livers at 0.25, 0.5, and 1 h after exposure to MC-LR compared with the control fish. *Indicates a significant difference between treatment and control.

The expression of GST gene was significantly decreased (*P* < 0.05) after injection of MC-LR for 0.25 h (Figure 1).

### RNA-seq and *de-novo* assembly

**Figure 2:**
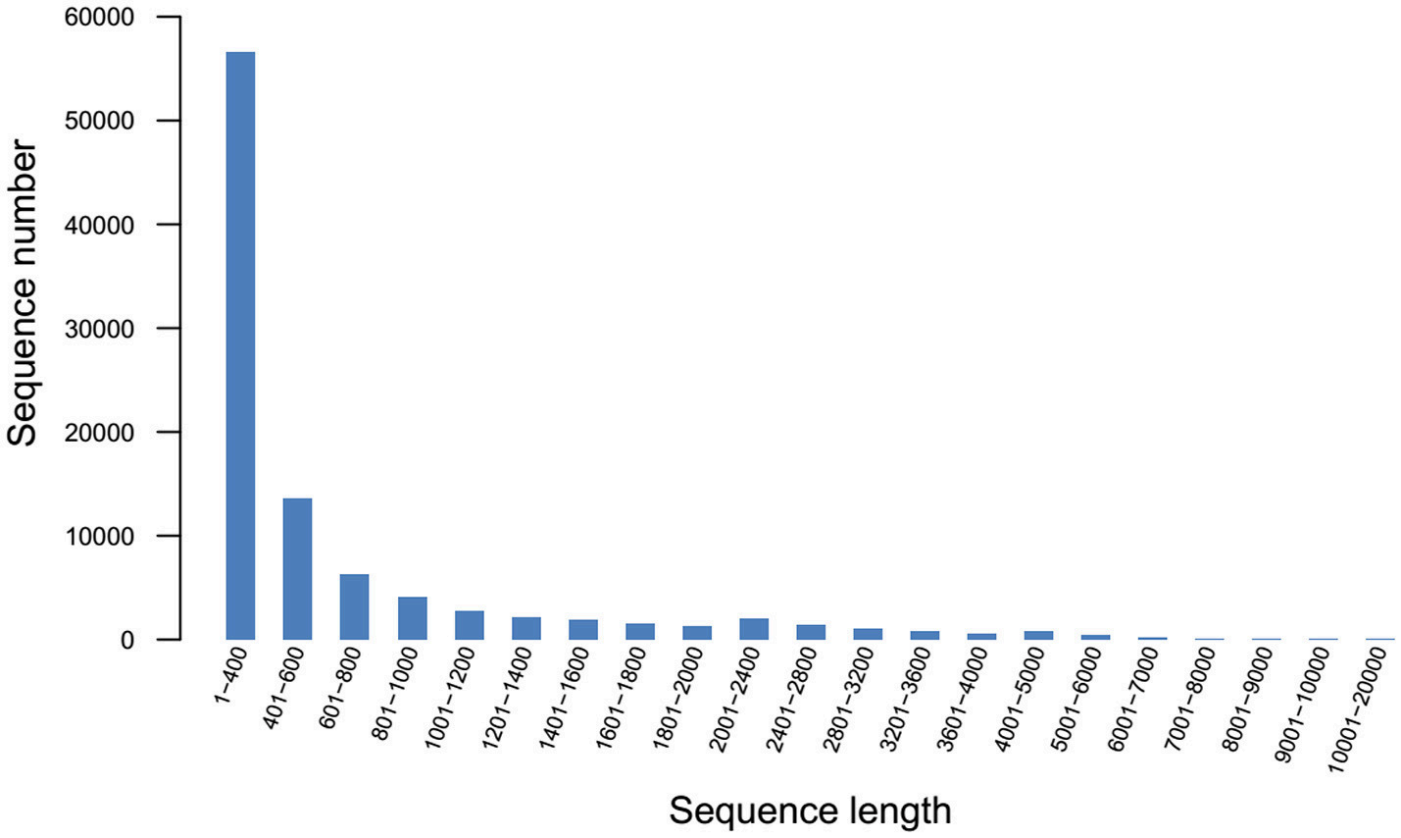
Unigene length distribution.

**Figure 3:**
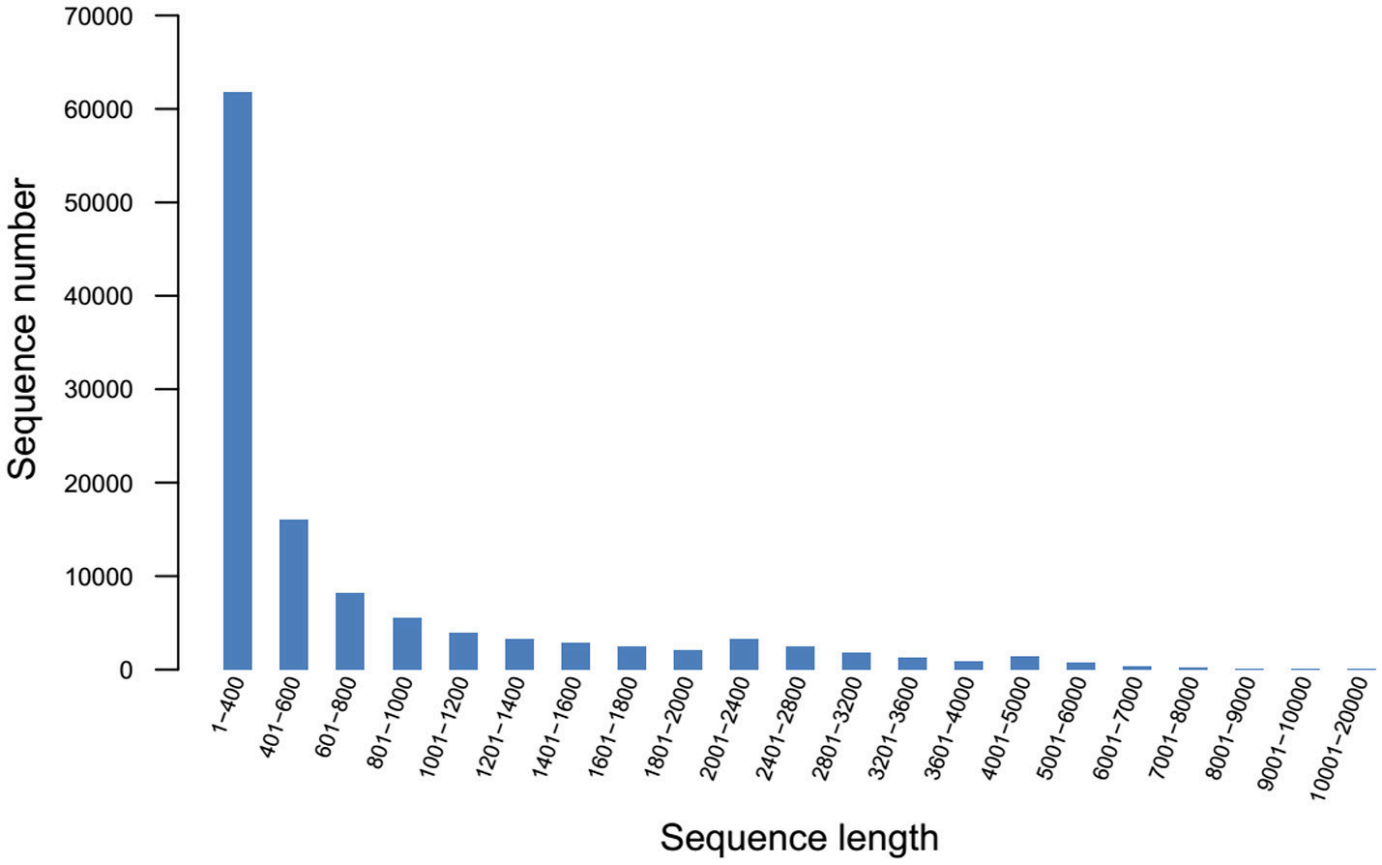
Transcript length distribution.

Q20 and Q30 values were all >90%, the base error rate was < 0.1%, and the percentage of GC content was similar, indicating that the data was accurate and reliable. A total of 97,505 unigenes (Figure 2) and 118,832 transcripts (Figure 3) were obtained by sequencing.

### Gene annotations

**Figure 4:**
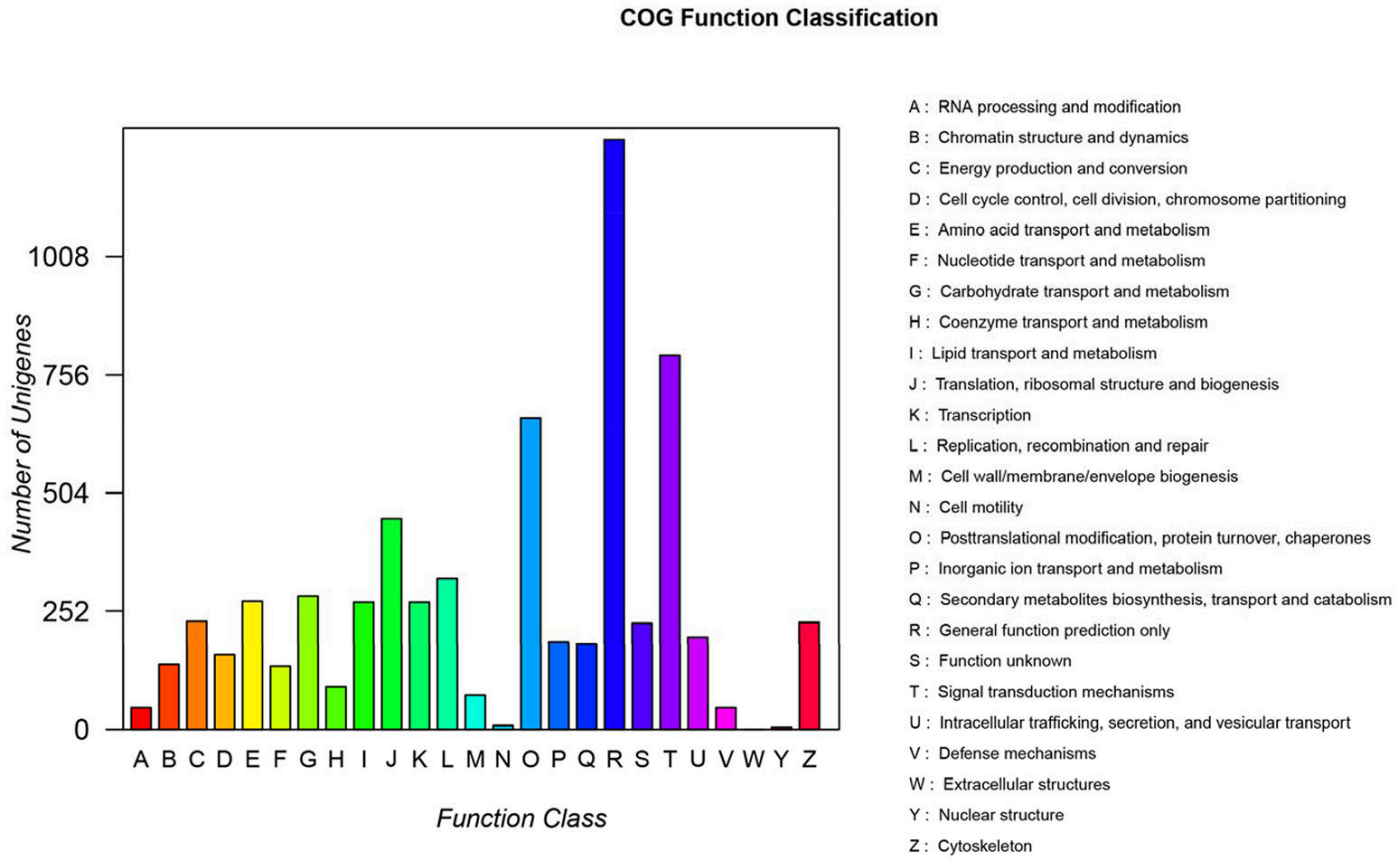
COG function classification. A, RNA processing and modification; B, Chromatin structure and dynamics; C, Energy production and conversion; D, Cell cycle control, cell division, chromosome partitioning; E, Amino acid transport and metabolism; F, Nucleotide transport and metabolism; G, Carbohydrate transport and metabolism; H, Coenzyme transport and metabolism; I, Lipid transport and metabolism; J, Translation and ribosomal structure and biogenesis; K, Transcription; L, Replication, recombination, and repair; M, Cell wall/membrane/envelope biogenesis; N, Cell motility; O, Posttranslational modification, protein turnover, and chaperones; P, Inorganic ion transport and metabolism; Q, Secondary metabolite biosynthesis, transport, and catabolism; R, General function prediction only; S, Function unknown; T, Signal transduction mechanisms; U, Intracellular trafficking, secretion, and vesicular transport; V, Defense mechanisms; W, Extracellular structures; Y, Nuclear structure; Z, Cytoskeleton.

Of the 97,505 unigenes obtained by RNA-seq, 14,614 unigenes were annotated into 25 COG terms. Among them, the number of unigenes that clustered into general function prediction was the highest (1,249), followed by signal transduction mechanisms (766) and replication, and recombination and repair (301). The number of unigenes with unknown function was 208 (Figure 4).

**Figure 5:**
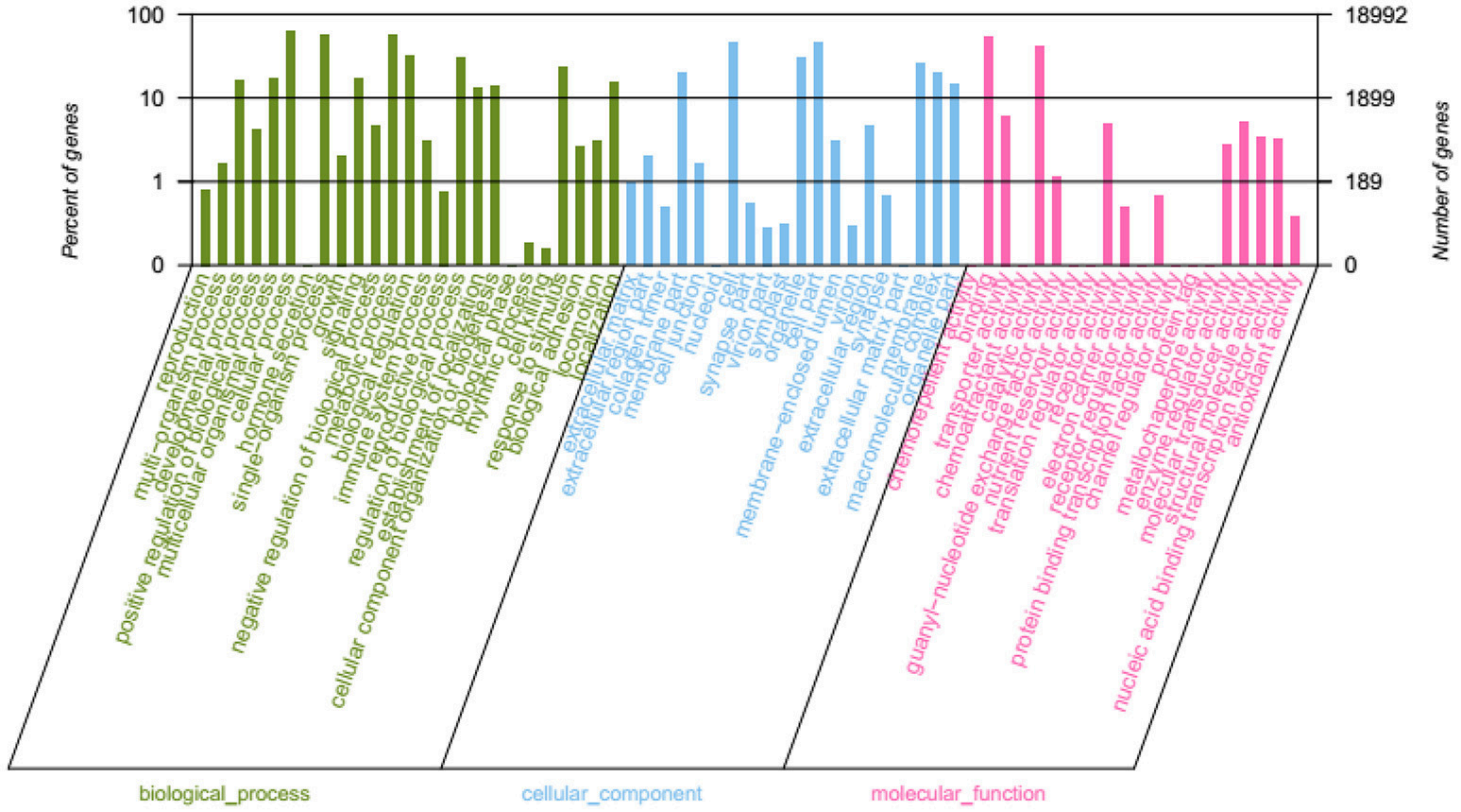
GO function classification.

Through GO terms, 71,192 unigenes were annotated to the biological process categories (Figure 5), which were divided into 25 sub categories, including metabolic process, developmental process, hormone secretion, and response to stimulus. A total of 40,767 unigenes were annotated into the cellular component categories, which were divided into 20 sub categories, including extracellular matrix, cell junction, and macromolecular complex. Furthermore, 23,351 unigenes were annotated to the molecular function class, which were divided into 20 sub categories, including antioxidant activity, receptor activity, and electron carrier activity.

**Figure 6:**
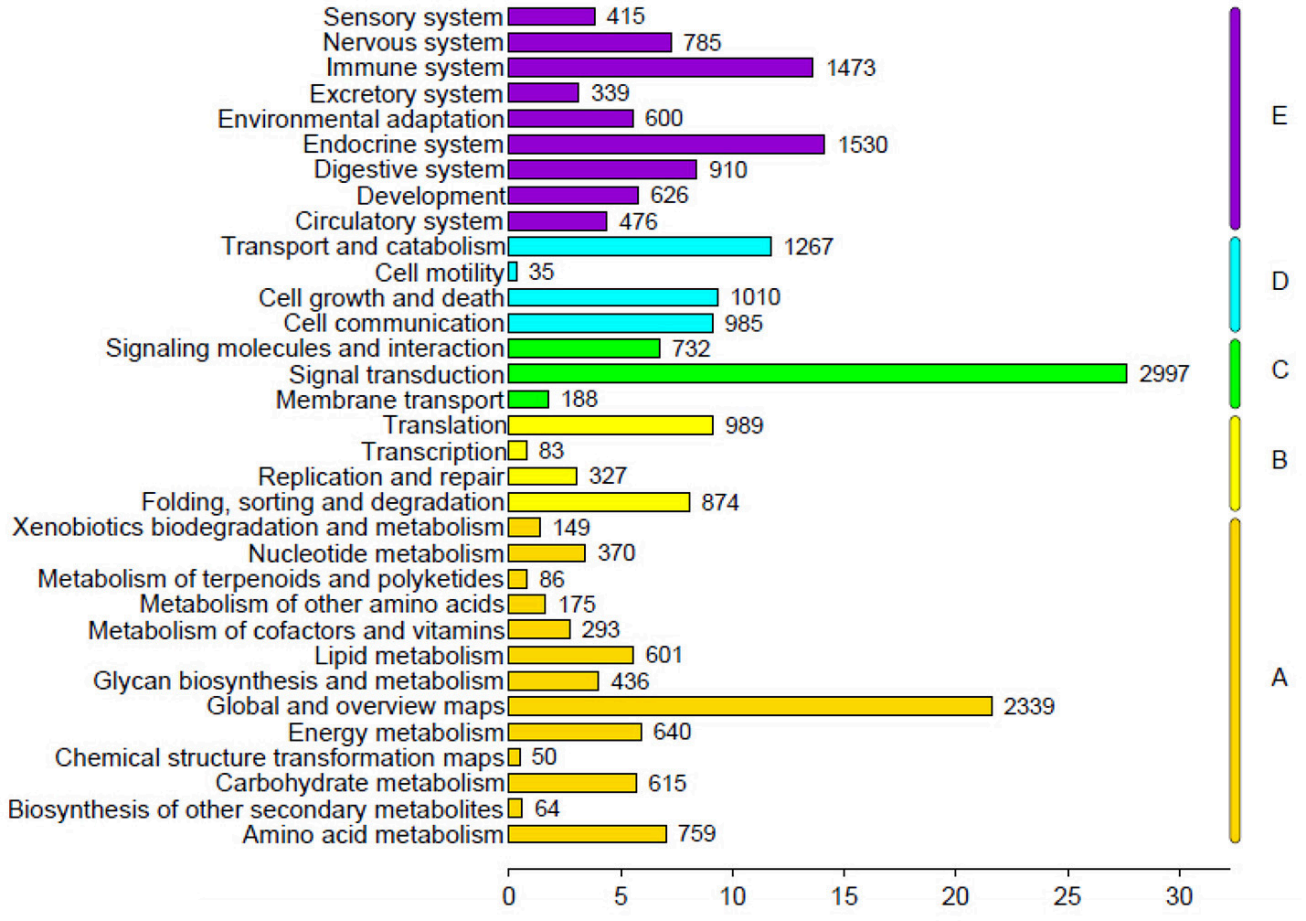
KEGG pathway classification.

The genes were classified according to the KEGG pathway (Figure 6) and divided into five branches: metabolism, genetic information processing, environmental information processing, cellular processes, and organismal systems. A total of 23,218 unigenes were annotated into 33 subclasses of the KEGG database, of which the number of unigenes in signal transduction was the largest (2,997), followed by global and overview maps (2,339) and endocrine system (1,530).

### Analysis of DEGs

**Figure 7:**
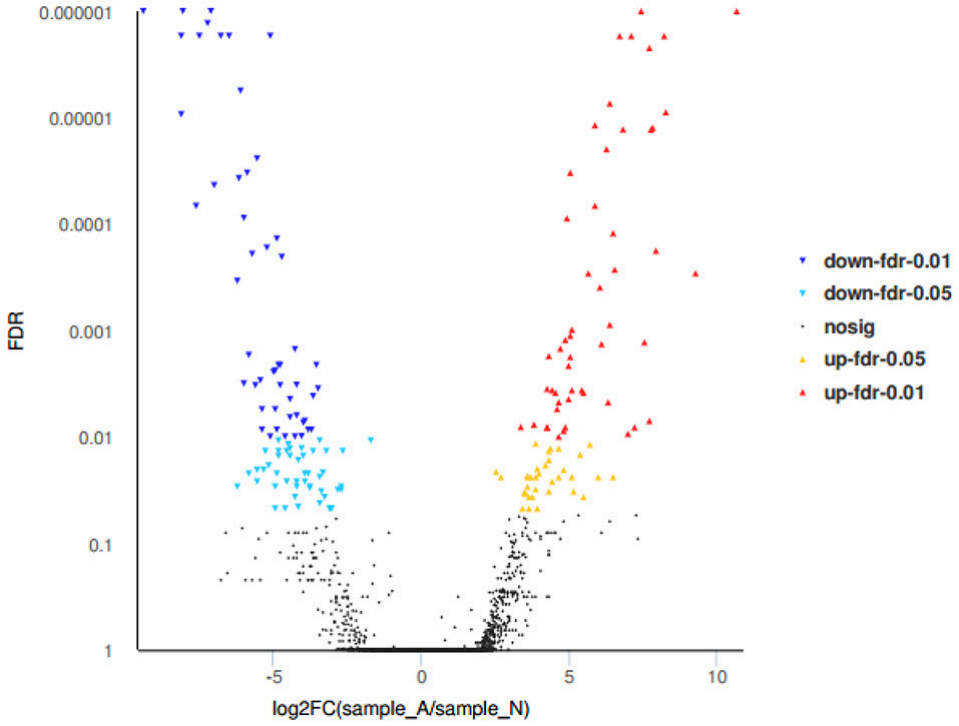
Volcano plots of differentially expressed genes.

RNA-seq revealed that the liver tissue contained 97,505 unigenes and 26,960 differentially expressed unigenes, with 184 unigenes showing significant differences, of which 86 were significantly increased, 98 were significantly decreased, and 75 unknown genes also found (Figure 7).

**Figure 8:**
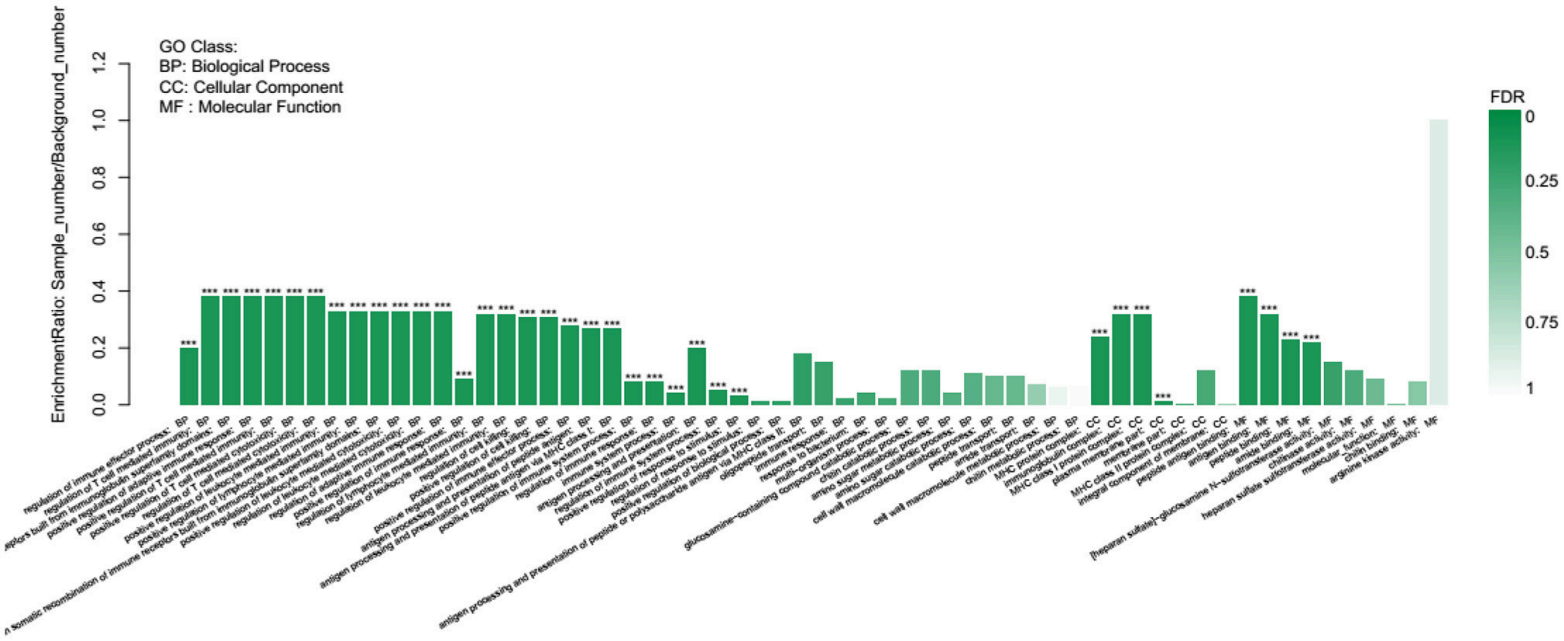
GO enrichment analysis of differentially expressed genes.

GO term enrichment analysis showed that 35 of the 145 GO terms enriched were significantly enriched. The differential genes are mainly enriched in 80 biological processes, mainly including the regulation of immune effector process, positive regulation of adaptive immune response, and regulation of response to stimulus, of which the immune-related GO term accounted for the most. The cellular components were mainly enriched in 19 terms, such as MHC protein complex, immunoglobulin complex, and plasma membrane part. From the molecular function of the DEGs, antigen binding, peptide binding, and amide binding were mainly enriched in 46 terms (Figure 8).

**Figure 9:**
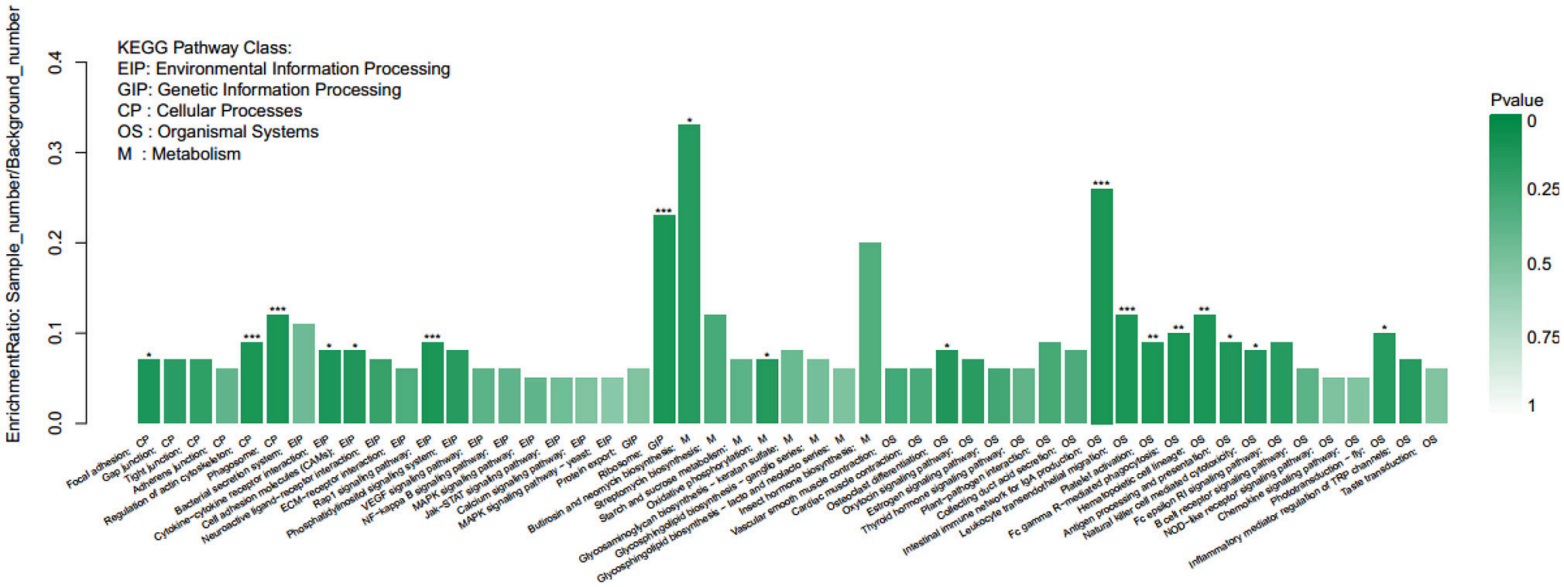
KEGG enrichment analysis of differentially expressed genes.

KEGG pathway enrichment analysis demonstrated that 18 of the 189 pathways were significantly enriched, and the most significant was a ribosome pathway that contains 77 differentially expressed genes, followed by the phagosome, comprising 43 genes. The pathways associated with this study are glutathione metabolism, peroxisome, oxidative phosphorylation, cytokine-cytokine receptor interaction, fatty acid metabolism, and apoptosis (Figure 9).

### miRNA-seq

miRNA-seq showed that miRNA length was mainly distributed in the range of 20–23 nt, with 22 nt sequences being the largest, followed by 21 and 23 nt.

**Figure 10:**
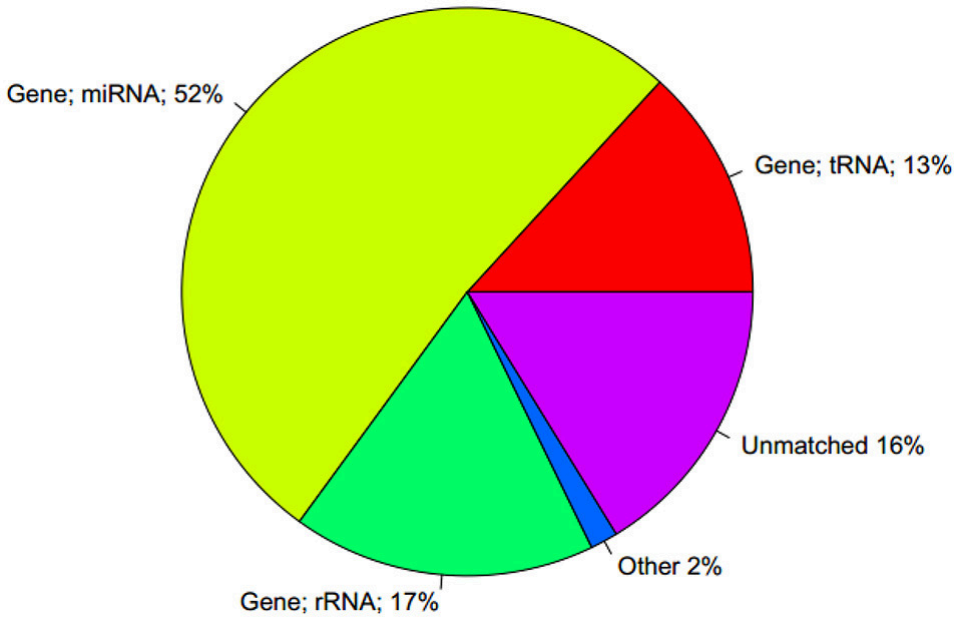
Comparison of the statistical findings of unique sequence and Rfam database.

Using the Rfam database to annotate the small RNAs, only 56% of the sequences were labeled as miRNA. In addition, 15 and 12% of the sequences were labeled as rRNA and tRNA, respectively (Figure 10).

### Expression of known and novel miRNAs and prediction of their target genes

A total of 286 known miRNAs were obtained by high-throughput sequencing, and 332 miRNA precursor sequences were predicted. Among them, only 11 miRNAs were expressed in the exposure group, including dre-miR-10b-3p, dre-miR-132-5p, dre-miR-181c-3p, dre-miR-193b-5p, dre-miR-196d, dre-miR-204-3p, dre-miR-23a-5p, dre-miR-34b, dre-miR-7146-3p, dre-miR-723-5p, and dre-miR-726. Only 15 miRNAs were expressed in the control group, including dre-miR-10d-3p, dre-miR-1306, dre-miR-133a-2-5p, dre-miR-133a-5p, dre-miR-137-3p, dre-miR-139-3p, dre-miR-15b-3p, dre-miR-1788-3p, dre-miR-182-3p, dre-miR-206-3p, dre-miR-20b-3p, dre-miR-34c-3p, dre-miR-430a-5p, dre-miR-728, and dre-miR-738. However, the expression levels of these miRNAs were relatively low.

In the 438 new miRNAs predicted in this experiment, only 3 were expressed in the exposure group, including 16_12512, 14_10184, and 15_11934, whereas only 5 were expressed in the control group, including 15_10997, 4_34750, 9_45354, 5_36269, and 10_3104. In addition, the top 10 new miRNAs with high expression levels were 10_2618, 14_9339, 24_28815, 9_44984, 29494, 10_3379, 15_10640, 15_10639, 25_29266, and 11_5017.

Among the 286 known miRNAs, 200 showed significant changes in expression levels, of which 138 were significantly increased and 62 were significantly decreased. Among the 438 new miRNAs identified, 281 expressed significant differences, of which 179 were significantly increased and 102 were significantly decreased.

A total of 104,8575 mRNA-miRNA interaction sites were obtained by target gene prediction from 286 known miRNAs and 438 new miRNAs. In addition, 21,252 and 21,241 target genes were respectively predicted in known and new miRNAs.

### The verification of some RNA-seq data by qPCR

**Figure 11:**
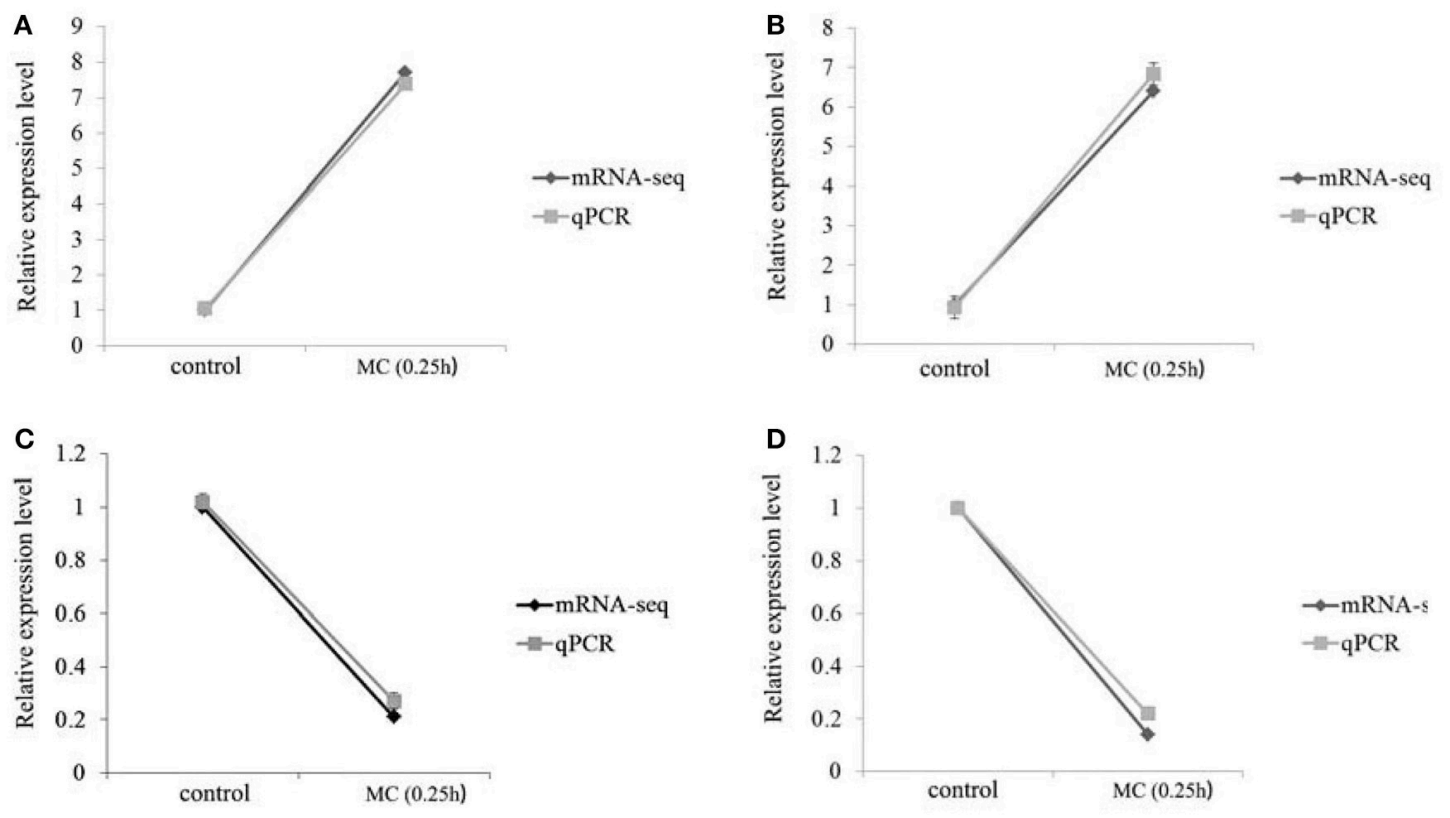
Comparative analysis of mRNA-seq and qPCR on four genes. **(A)** HO-1; **(B)** IL-10R1; **(C)** apoA-I; **(D)** HBP2.

The expressions of HO-1 and IL-10R1 in the toxic group at 0.25 h were significantly increased, whereas the expressions of HBP2 and apoA-1 were significantly decreased. The RNA results were consistent with the qPCR findings. Accordingly, the reliability and accuracy of the RNA-seq data were confirmed (Figure 11).

**Figure 12:**
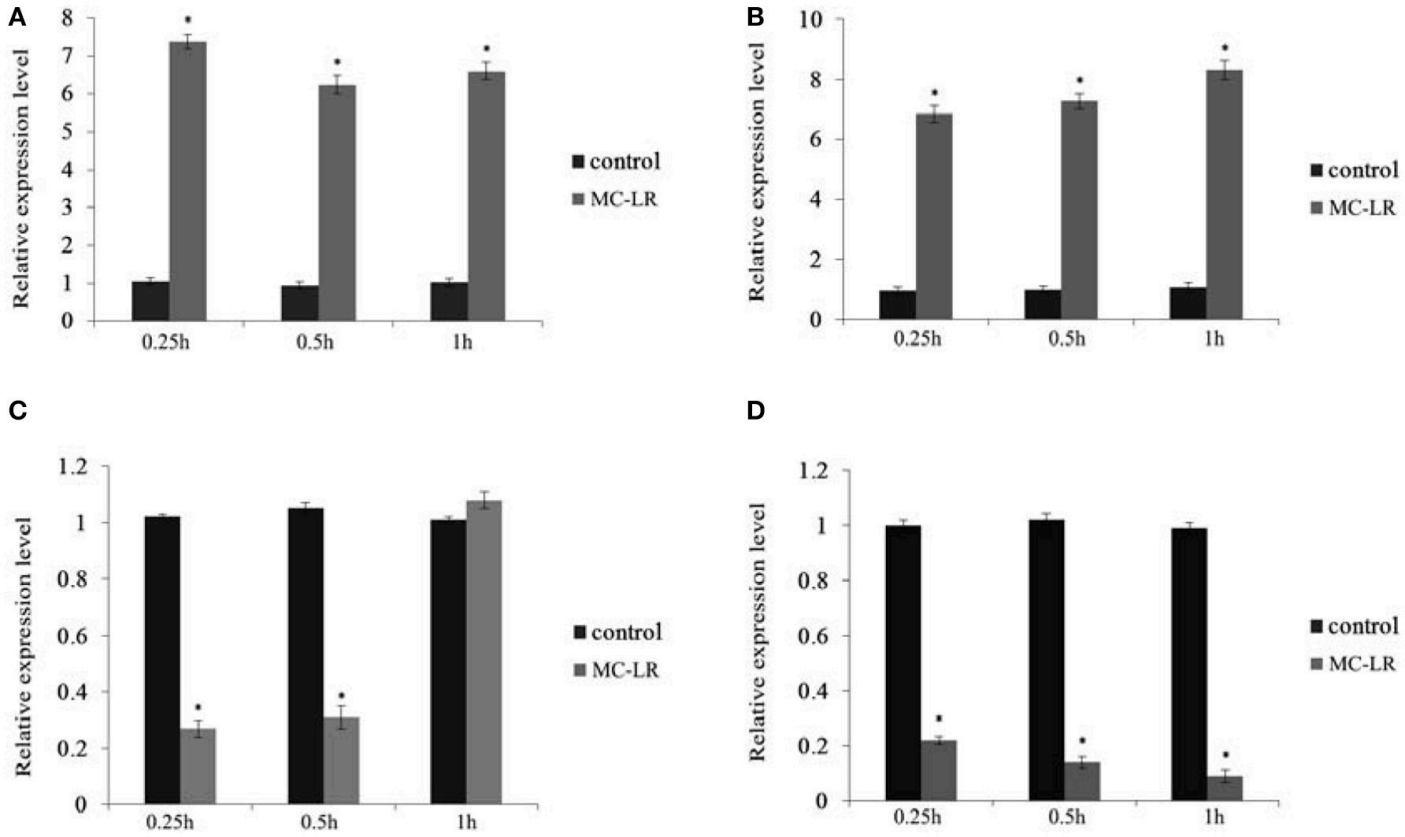
Expression of four genes in 0.25, 0.5, and 1 h samples. **(A)** HO-1; **(B)** IL-10R1; **(C)** apoA-I; **(D)** HBP2. *Indicates a significant difference between treatment and control.

The expression of HO-1 gene was significantly increased at 0.25 h after injection of MC-LR and was significantly higher than that of control group at 0.5 and 1 h. IL-10R1 gene expressions at three time points significantly increased. The expression level of apoA-I gene was significantly decreased 0.25 and 0.5 h after injection, but the expression significantly exceeded the control level at 1 h. HBP2 gene expression after injection showed a sustained decline with a significant difference compared to the control (Figure 12).

## Discussion

The GST gene in the liver tissue of silver carp began to play a role in resisting MC-LR at 0.25 h. Therefore, samples from the exposure and control groups at 0.25 h were selected for RNA-seq and miRNA-seq.

Of the 97,505 unigenes obtained by transcriptome sequencing in this study, 184 were significantly different. Of these significantly different unigenes, 86 were significantly increased, 98 were significantly decreased, and 75 were unknown. Transcriptome analysis showed that GST gene expression significantly decreased, and this observation was consistent with the results of semi-quantitative PCR. GO enrichment analysis revealed that 35 GO terms were significantly enriched, and the different gene was enriched in the biological process and immune-related term, indicating that the immune system of fish injected with MC-LR resists hepatocyte inflammation and damage. KEGG pathway analysis demonstrated that glutathione metabolism pathway, oxidative phosphorylation pathway, cytokine and receptor interaction pathway, MAPK signal pathway, and apoptosis pathway are related to MC-LR toxicity mechanism and detoxification. miRNA sequencing identified 286 known miRNAs, predicted 332 miRNA precursor sequences and 438 new miRNAs, and detected 1,048,575 mRNA–miRNA interaction sites through the target gene prediction of miRNAs.

Heme oxygenase 1 (HO-1) belongs to the heme oxygenase-induced type. When an organism undergoes oxidative stress, inflammatory reaction, hypoxia, and toxin invasion, HO-1 is induced to resist damage. HO-1 participates in various reactions induced by external stimuli (Alam et al., 2004), keeps the balance of tissues and organs, prevents oxidative stress, and maintains cell integrity. Interleukin-10 receptor 1 (IL-10R1) is a well-known anti-inflammatory cytokine that plays an important role in immune responses, including immune cell activation and cytokine and chemokine production (Saraiva and O'Garra, 2010). IL-10R has two subtypes, namely, IL-10R1 and IL-10R2. IL-10R1 belongs to IL-10 receptor-specific part, which is involved in IL-10 signaling pathway. Apolipoprotein A-I (apoA-I) is a major protein component of high-density lipoprotein, with many immune-related properties, including bacterial endotoxin inhibition, antiviral activity, and inflammatory factor inhibition. Heme binding protein 2 (HBP2) exhibits a high affinity to hemoglobin and performs unique functions in physiological metabolism, anti-inflammatory response, cell differentiation, and necrosis (Sato et al., 2004).

Among the target genes regulated by miRNA, four genes, namely, HO-1, IL-10R1, apoA-I, and HBP2 were used for qPCR verification. The results were consistent with the high-throughput data, and the accuracy of the sequencing technique was verified. HO-1 plays an important role in antioxidant, anti-inflammatory, and anti-apoptotic processes (Otterbein and Choi, 2000; Chung et al., 2009). Tzaneva and Perry (2014) showed that goldfish exposed to hypoxic state can increase its HO-1 enzyme activity and confirmed that HO-1 is involved in fish respiratory process. HO-1 gene is a key factor in cell resistance to oxidative stress and key element in maintaining cell integrity and viability. Voelker et al. (2008) studied the response of zebrafish embryos exposed to 13 different environmental pollutants by using microarray and found that HO-1 is a sensitive and widely induced gene that elicits cytoprotective effects (Voelker et al., 2008). MC-LR causes a significant increase in HO-1 expression in human hepatocellular carcinoma cells (Gan et al., 2010). In the current study, the expression of HO-1 significantly increased 0.25 h after MC-LR was injected, indicating that HO-1 helped protect cells. We speculated that HO-1, as a gene regulating heat shock protein, may protect cells before GST performs its functions because an increased HO-1 expression is considered a key cytoprotective effect when organisms undergo oxidative damage, pathologic inflammation, ischemia, and radiation (Choi and Alam, 1996; Dennery, 2000).

IL-10R1 belongs to the class II cytokine receptor family (CRF2). IL-10 signaling pathway plays its roles by closely binding to IL-10R1, thereby inducing changes in the IL-10 structure; IL-10 also binds to IL-10R2 and participates in the JAK-STAT signal transduction pathway (Kotenko et al., 1997). The inhibition of the IL-10R1 activity can prevent IL-10 gene expression. IL-10R1 expression can be detected when IL-10 functions, demonstrating that IL-10R1 is a specific part of IL-10 receptor and has an important part in the IL-10 signaling pathway (O'Farrell et al., 1998). In the current study, the expression of IL-10R1 gene in fish injected with MC-LR at 0.25, 0.5, and 1 h continuously increased, and the transcriptome data showed that the expression of IL-10 gene at 0.25 h significantly increased, indicating that the IL-10 gene activated the immune response against MC-induced inflammatory lesions.

Human apoA-I gene exhibits direct activity against bacteria and viruses, such as human immunodeficiency virus and xenotropic murine virus (Alonso-Villaverde et al., 2003). However, in fish, only rainbow trout, catfish, and striped bass possess the antibacterial activity of apoA-I gene as demonstrated in *in vitro* experiments (Villarroel et al., 2007; Johnston et al., 2008; Pridgeon and Klesius, 2013). Many studies on mammalian defense-related processes and components, such as antiviral substances, antibacterial, and anti-inflammatory activity, have focused on apoA-I genes (Srinivas et al., 1991; Tada et al., 1993; Burger and Dayer, 2002). ApoA-I participates in lipid transport and uptake, and most fish use lipids as their primary source of energy; thus, lipid metabolism is necessary to maintain internal environmental balance in fish (Kondo et al., 2005). In the current study, apoA-I gene expression significantly decreased 0.25 h after MC-LR injection, and MC-LR inhibited apoA-I gene expression. However, its expression increased after 0.5 h and reached levels higher than that of the control group after 1 h, suggesting that apoA-I gene exhibits an anti-inflammatory function.

HBP2 gene can induce NH3T3 cell necrosis through its specific role in the BH3 region (Ambrosi et al., 2011). HBP2 can promote mitochondrial permeability transition, resulting in cell death (Szigeti et al., 2006). HBP2 can also inhibit the anti-apoptotic protein Bcl-xL (Szigeti et al., 2010). However, the specific regulatory mechanism of HBP2 is unclear. In the current study, the expression of HBP2 significantly decreased at the three time points after MC-LR injection, indicating that HBP2 gene expression was inhibited by MC-LR. Although HBP2 binds to heme, limited information regarding its physiological functions is available.

Although some functional studies on the four genes have been performed, the functions of HO-1, IL-10R1, and HBP2, especially HBP2, in silver carp are unclear. However, the expression levels of the four genes are altered by MC-LR stimulation in fish and regulated by miRNA, indicating that the four genes are associated with the effects of MC on silver carp. In the current study, we combined transcriptome data with miRNA data and obtained a large amount of data regarding the toxic mechanism of MC-LR in silver carp and its detoxification. We also provided a basis for future studies on the toxic effects and mechanisms of MC in fish.

## Author contributions

QL and YW: Designed and led the study; MH, XQ, LP, and CF: Performed the experiments; MH, XQ, QL, and YW: Analyzed data; YS, MH and YW: Wrote the manuscript. All authors reviewed the manuscript.

### Conflict of interest statement

The authors declare that the research was conducted in the absence of any commercial or financial relationships that could be construed as a potential conflict of interest.

## References

[B1] Alam J. Igarashi K. Immenschuh S. Shibahara S. Tyrrell R. M. (2004). Regulation of heme oxygenase-1 gene transcription: recent advances and highlights from the International Conference (Uppsala, 2003) on Heme Oxygenase. Antioxid. Redox Signal. 6, 924–933. 10.1089/ars.2004.6.924 15345152

[B2] Alonso-Villaverde C. Segues T. Coll-Crespo B. Perez-Bernalte R. Rabassa A. Gomila M.. (2003). High-density lipoprotein concentrations relate to the clinical course of HIV viral load in patients undergoing antiretroviral therapy. AIDS 17, 1173–1178. 10.1097/00002030-200305230-00009 12819519

[B3] Ambros V. (2004). The functions of animal microRNAs. Nature 431, 350–355. 10.1038/nature02871 15372042

[B4] Ambrosi E. Capaldi S. Bovi M. Saccomani G. Perduca M. Monaco H. L. (2011). Structural changes in the BH3 domain of SOUL protein upon interaction with the anti-apoptotic protein Bcl-xL. Biochem. J. 438, 291–301. 10.1042/BJ20110257 21639858 PMC3174058

[B5] Amrani A. Nasri H. Azzouz A. Kadi Y. Bouaicha N. (2014). Variation in cyanobacterial hepatotoxin (microcystin) content of water samples and two species of fishes collected from a shallow lake in Algeria. Arch. Environ. Contam. Toxicol. 66, 379–389. 10.1007/s00244-013-9993-2 24445842

[B6] Bian L. Liu C. Chen S. Zhao F. Ge J. Tan J. (2017). Transcriptome analysis of gene expression patterns during embryonic development in golden cuttlefish (*Sepia esculenta*). Genes Genomics 40, 253–263. 10.1007/s13258-017-0588-6 29892796

[B7] Burge S. W. Daub J. Eberhardt R. Tate J. Barquist L. Nawrocki E. P.. (2013). Rfam 11.0: 10 years of RNA families. Nucleic Acids Res. 41, D226–D232. 10.1093/nar/gks1005 23125362 PMC3531072

[B8] Burger D. Dayer J. M. (2002). High-density lipoprotein-associated apolipoprotein A-I: the missing link between infection and chronic inflammation? Autoimmun. Rev. 1, 111–117. 10.1016/S1568-9972(01)00018-0 12849067

[B9] Chen Y. M. Lee T. H. Lee S. J. Huang H. B. Huang R. Chou H. N. (2006). Comparison of protein phosphatase inhibition activities and mouse toxicities of microcystins. Toxicon 47, 742–746. 10.1016/j.toxicon.2006.01.026 16684551

[B10] Choi A. M. Alam J. (1996). Heme oxygenase-1: function, regulation, and implication of a novel stress-inducible protein in oxidant-induced lung injury. Am. J. Respir. Cell Mol. Biol. 15, 9–19. 10.1165/ajrcmb.15.1.8679227 8679227

[B11] Chung S. W. Hall S. R. Perrella M. A. (2009). Role of haem oxygenase microbial host defence. Cell. Microbiol. 11, 199–207. 10.1111/j.1462-5822.2008.01261.x 19016784 PMC3080039

[B12] Dennery P. A. (2000). Regulation and role of heme oxygenase in oxidative injury. Curr. Top. Cell. Regul. 36, 181–199. 10.1016/S0070-2137(01)80008-X 10842752

[B13] Dhakshinamoorthy S. Long D. J. II. Jaiswal A. K. (2000). Antioxidant regulation of genes encoding enzymes that detoxify xenobiotics and carcinogens. Curr. Top. Cell. Regul. 36, 201–216. 10.1016/S0070-2137(01)80009-1 10842753

[B14] Francis G. (1978). Poisonous Australian Lake. Nature 18, 11–12. 10.1038/018011d0

[B15] Friedländer M. R. Mackowiak S. D. Li N. Chen W. Rajewsky N. (2012). miRDeep2 accurately identifies known and hundreds of novel microRNA genes in seven animal clades. Nucleic Acids Res. 40, 37–52. 10.1093/nar/gkr688 21911355 PMC3245920

[B16] Gan N. Sun X. Song L. (2010). Activation of Nrf2 by microcystin-LR provides advantages for liver cancer cell growth. Chem. Res. Toxicol. 23, 1477–1484. 10.1021/tx1001628 20722399

[B17] Goldstein J. A. Faletto M. B. (1993). Advances in mechanisms of activation and deactivation of environmental chemicals. Environ. Health Perspect. 100, 169–176. 10.1289/ehp.93100169 8354165 PMC1519589

[B18] Guo L. (2007). Doing battle with the green monster of Taihu Lake. Science 317:1166. 10.1126/science.317.5842.1166 17761862

[B19] Ha J. H. Hidaka T. Tsuno H. (2009). Quantification of toxic *Microcystis* and evaluation of its dominance ratio in blooms using real-time PCR. Environ. Sci. Technol. 43, 812–818. 10.1021/es801265f 19245020

[B20] Höck J. Meister G. (2008). The argonaute protein family. Genome Biol. 9:210. 10.1186/gb-2008-9-2-210 18304383 PMC2374724

[B21] Hoeger S. J. Hitzfeld B. C. Dietrich D. R. (2005). Occurrence and elimination of cyanobacterial toxins in drinking water treatment plants. Toxicol. Appl. Pharmacol. 203, 231–242. 10.1016/j.taap.2004.04.015 15737677

[B22] Huang S. Cao X. Tian X. Wang W. (2016). High-throughput sequencing identifies MicroRNAs from posterior intestine of loach (*Misgurnus anguillicaudatus*) and their response to intestinal air-breathing inhibition. PLoS ONE 11:e0149123. 10.1371/journal.pone.0149123 26872032 PMC4752256

[B23] Itoh K. Ishii T. Wakabayashi N. Yamamoto M. (1999). Regulatory mechanisms of cellular response to oxidative stress. Free Radic. Res. 31, 319–324. 10.1080/10715769900300881 10517536

[B24] Johnston L. D. Brown G. Gauthier D. Reece K. Kator H. Van Veld P. (2008). Apolipoprotein A-I from striped bass (*Morone saxatilis*) demonstrates antibacterial activity *in vitro*. Comp. Biochem. Physiol. B Biochem. Mol. Biol. 151, 167–175. 10.1016/j.cbpb.2008.06.011 18627791

[B25] Kim Y. K. Yu J. Han T. S. Park S. Y. Namkoong B. Kim D. H.. (2009). Functional links between clustered microRNAs: suppression of cell-cycle inhibitors by microRNA clusters in gastric cancer. Nucleic Acids Res. 37, 1672–1681. 10.1093/nar/gkp002 19153141 PMC2655672

[B26] Kondo H. Morinaga K. Misaki R. Nakaya M. Watabe S. (2005). Characterization of the pufferfish *Takifugu rubripes* apolipoprotein multigene family. Gene 346, 257–266. 10.1016/j.gene.2004.11.015 15716036

[B27] Kotenko S. V. Krause C. D. Izotova L. S. Pollack B. P. Wu W. Pestka S. (1997). Identification and functional characterization of a second chain of the interleukin-10 receptor complex. EMBO J. 16, 5894–5903. 10.1093/emboj/16.19.5894 9312047 PMC1170220

[B28] Lee R. C. Feinbaum R. L. Ambros V. (1993). The C. elegans heterochronic gene *lin-4* encodes small RNAs with antisense complementarity to lin-14. Cell 75, 843–854. 10.1016/0092-8674(93)90529-Y 8252621

[B29] Li H. Durbin R. (2009). Fast and accurate short read alignment with Burrows-Wheeler transform. Bioinformatics 25, 1754–1760. 10.1093/bioinformatics/btp324 19451168 PMC2705234

[B30] Magalhães V. F. Soares R. M. Azevedo S. M. (2001). Microcystin contamination in fish from the Jacarepagua Lagoon (Rio de Janeiro, Brazil): ecological implication and human health risk. Toxicon 39, 1077–1085. 10.1016/S0041-0101(00)00251-8 11223098

[B31] Martins N. D. Yunes J. S. Monteiro D. A. Rantin F. T. Kalinin A. L. (2017). Microcystin-LR leads to oxidative damage and alterations in antioxidant defense system in liver and gills of *Brycon amazonicus* (spix & agassiz, 1829). Toxicon 139, 109–116. 10.1016/j.toxicon.2017.10.006 29024772

[B32] Miska E. A. (2005). How microRNAs control cell division, differentiation and death. Curr. Opin. Genet. Dev. 15, 563–568. 10.1016/j.gde.2005.08.005 16099643

[B33] Nishizawa T. Ueda A. Asayama M. Fujii K. Harada K. Ochi K.. (2000). Polyketide synthase gene coupled to the peptide synthetase module involved in the biosynthesis of the cyclic heptapeptide microcystin. J. Biochem. 127, 779–789. 10.1093/oxfordjournals.jbchem.a022670 10788786

[B34] O'Farrell A. M. Liu Y. Moore K. W. Mui A. L. (1998). IL-10 inhibits macrophage activation and proliferation by distinct signaling mechanisms: evidence for Stat3-dependent and -independent pathways. EMBO J. 17, 1006–1018. 10.1093/emboj/17.4.1006 9463379 PMC1170450

[B35] Otterbein L. E. Choi A. M. (2000). Heme oxygenase: colors of defense against cellular stress. Am. J. Physiol. Lung Cell. Mol. Physiol. 279, L1029–L1037. 10.1152/ajplung.2000.279.6.L1029 11076792

[B36] Pouria S. De-Andrade A. Cavalcanti J. R. Barreto V. Ward C. Preiser W.. (1998). Fatal microcystin intoxication in haemodialysis unit in Caruaru, Brazil. Lancet 352, 21–26. 10.1016/S0140-6736(97)12285-1 9800741

[B37] Pridgeon J. W. Klesius P. H. (2013). Apolipoprotein A channel catfish: transcriptional analysis, antimicrobial activity, and efficacy as plasmid DNA immunostimulant against *Aeromonas hydrophila* infection. Fish Shellfish Immunol. 35, 1129–1137. 10.1016/j.fsi.2013.07.028 23954697

[B38] Qu X. Zhang K. Cui Z. Zhang Y. Jiang J. Feng L.. (2011). Construction and analysis of liver suppression subtractive hybridization library of silver carp (*Hypophthalmichthys Molitrix*) intraperitoneally injected with Microcystin-LR. Aquat. Toxicol. 105, 151–156. 10.1016/j.aquatox.2011.06.005 21718658

[B39] Saraiva M. O'Garra A. (2010). The regulation of IL-10 production by immune cells. Nat. Rev. Immunol. 10, 170–181. 10.1038/nri2711 20154735

[B40] Sato E. Sagami I. Uchida T. Sato A. Kitagawa T. Igarashi J.. (2004). SOUL in mouse eyes is a new hexameric heme-binding protein with characteristic optical absorption, resonance Raman spectral, and heme-binding properties. Biochemistry 43, 14189–14198. 10.1021/bi048742i 15518569

[B41] Song W. Jiang K. Zhang F. Lin Y. Ma L. (2015). Transcriptome sequencing, *De Novo* assembly and differential gene expression analysis of the early development of *Acipenser baeri*. PLoS ONE 10:e0137450. 10.1371/journal.pone.0137450 26359664 PMC4567377

[B42] Srinivas R. V. Venkatachalapathi Y. V. Rui Z. Owens R. J. Gupta K. B. Srinivas S. K.. (1991). Inhibition of virus-induced cell fusion by apolipoprotein A-I and its amphipathic peptide analogs. J. Cell. Biochem. 45, 224–237. 10.1002/jcb.240450214 1647394

[B43] Sykora J. L. Keleti G. (1981). Cyanobacteria and Endotoxins in Drinking Water Supplies, in The Water Environment. Environmental Science Research, ed Carmichael W. W. (Boston, MA: Springer).

[B44] Szigeti A. Bellyei S. Gasz B. Boronkai A. Hocsak E. Minik O.. (2006). Induction of necrotic cell death and mitochondrial permeabilization by heme binding protein 2/SOUL. FEBS Lett. 580, 6447–6454. 10.1016/j.febslet.2006.10.067 17098234

[B45] Szigeti A. Hocsak E. Rapolti E. Racz B. Boronkai A. Pozsgai E.. (2010). Facilitation of mitochondrial outer and inner membrane permeabilization and cell death in oxidative stress by a novel Bcl-2 homology 3 domain protein. J. Biol. Chem. 285, 2140–2151. 10.1074/jbc.M109.015222 19901022 PMC2804370

[B46] Tada N. Sakamoto T. Kagami A. Mochizuki K. Kurosaka K. (1993). Antimicrobial activity of lipoprotein particles containing apolipoprotein Al. Mol. Cell. Biochem. 119, 171–178. 10.1007/BF00926868 8455579

[B47] Tzaneva V. Perry S. F. (2014). Heme oxygenase-1 (HO-1) mediated respiratory responses to hypoxia in the goldfish, Carassius auratus. Respir. Physiol. Neurobiol. 199, 1–8. 10.1016/j.resp.2014.04.006 24780551

[B48] Ueno Y. Nagata S. Tsutsumi T. Hasegawa A. Watanabe M. F. Park H. D.. (1996). Detection of microcystins, a blue-green algal hepatotoxin, in drinking water sampled in Haimen and Fusui, endemic areas of primary liver cancer in China, by highly sensitive immunoassay. Carcinogenesis 17, 1317–1321. 10.1093/carcin/17.6.1317 8681449

[B49] Villarroel F. Bastias A. Casado A. Amthauer R. Concha M. I. (2007). Apolipoprotein A-I, an antimicrobial protein in *Oncorhynchus mykiss*: evaluation of its expression in primary defence barriers and plasma levels in sick and healthy fish. Fish Shellfish Immunol. 23, 197–209. 10.1016/j.fsi.2006.10.008 17391986

[B50] Voelker D. Stetefeld N. Schirmer K. Scholz S. (2008). The role of cyp and heme oxygenase 1 gene expression for the toxicity of 3,4-dichloroaniline in zebrafish (*Danio rerio*) embryos. Aquat. Toxicol. 86, 112–120. 10.1016/j.aquatox.2007.10.007 18045703

[B51] Wang Y. Xiu Y. Bi K. Ou J. Gu W. Wang W.. (2017). Integrated analysis of mRNA-seq in the haemocytes of *Eriocheir sinensis* in response to *Spiroplasma eriocheiris* infection. Fish Shellfish Immunol. 68, 289–298. 10.1016/j.fsi.2017.07.036 28734969

[B52] Wiegand C. Pflugmacher S. (2005). Ecotoxicological effects of selected cyanobacterial secondary metabolites: a short review. Toxicol. Appl. Pharmacol. 203, 201–218. 10.1016/j.taap.2004.11.002 15737675

[B53] Xie C. Mao X. Huang J. Ding Y. Wu J. Dong S.. (2011). KOBAS 2.0: a web server for annotation and identification of enriched pathways and diseases. Nucleic Acids Res. 39, W316–W322. 10.1093/nar/gkr483 21715386 PMC3125809

[B54] Xie Z. Z. Ling X. Dengdong W. Chao F. Qiongyu L. Zihao L. (2014). Transcriptome analysis of the *Trachinotus ovatus*: identification of reproduction, growth and immune-related genes and microsatellite markers. PLoS ONE 9:e109419 10.1371/journal.pone.0109419 25303650 PMC4193775

[B55] Xu X. Shen Y. Fu J. Lu L. Li J. (2014). *De novo* assembly of the grass carp *Ctenopharyngodon idella* transcriptome to identify miRNA targets associated with motile aeromonad septicemia. PLoS ONE 9:e112722. 10.1371/journal.pone.0112722 25409340 PMC4237362

[B56] Yi C. Guo L. Ni L. Luo C. (2016). Silver carp exhibited an enhanced ability of biomanipulation to control cyanobacteria bloom compared to bighead carp in hypereutrophic lake taihu mesocosms. Ecol. Eng. 89, 7–13. 10.1016/j.ecoleng.2016.01.022

[B57] Yoshizawa S. Matsushima R. Watanabe M. F. Harada K. Ichihara A. Carmichael W. W.. (1990). Inhibition of protein phosphatases by microcystins and nodularin associated with hepatotoxicity. J. Cancer Res. Clin. Oncol. 116, 609–614. 10.1007/BF01637082 2174896 PMC12200272

[B58] Zhang D. Xie P. Liu Y. Qiu T. (2009). Transfer, distribution and bioaccumulation of microcystins in the aquatic food web in Lake Taihu, China, with potential risks to human health. Sci. Total Environ. 407, 2191–2199. 10.1016/j.scitotenv.2008.12.039 19185334

